# The Maimonides Model for a Regimen of Health: A Comparison with the Contemporary Scenario

**DOI:** 10.5041/RMMJ.10396

**Published:** 2020-10-14

**Authors:** Isidor Segal, Shraga Blazer

**Affiliations:** 1 Emeritus Professor, Consulting Physician, Retired: “GIT Unit” Prince of Wales Hospital, Sydney, Australia; 2 Editor-in-Chief, Rambam Maimonides Medical Journal, Rambam Health Care Campus, Haifa, Israel

**Keywords:** Dietary recommendations, Maimonides, nutrition

## Abstract

Rabbi Moses Ben Maimon, known as Maimonides, or The “Rambam” (a Hebrew acronym for his name), was one of the greatest arbiters of all times on matters of Jewish law, one of the greatest philosophers of the Middle Ages, a scientist, and a researcher. In addition, he was a court physician to the Egyptian Sultan. In addition to his monumental work on Jewish law and ethics, his writings on medicine have been considered classics over the generations. The aim of this paper is to assess Maimonides’ health regimen and to compare his dietary recommendations with contemporary dietary regimens. To this end, Maimonides’ recommendations were compared to the modern guidelines of the United States, the Netherlands, and the World Health Organization (WHO), as well as to the Mediterranean diet, which is popular worldwide. Both marked similarities and contrasts were noted between Maimonides’ and modern recommendations. Most of Maimonides’ medical recommendations remain relevant more than 800 years later.

## INTRODUCTION

Rabbi Moses Maimonides, the Rambam, was born in Cordoba, Spain on March 30th, 1135.^1^ Maimonides was the spiritual leader of the Jewish people of his time, and his Halachic works and recommendations have become integral to modern-day Judaism. In addition, he was a pre-eminent physician and would eventually become the personal physician to the son of Saladin, al-Malik al-Afdal, the Sultan of Cairo. Although Maimonides only devoted time to medical writing during the last two decades of his life, he impacted the philosophy and medical practice of both the European and Arab cultures of his time.

A brief overview of his background is critical to understanding the depth and breadth of his medical knowledge and its impact on medicine in his time. At the age of 10, Maimonides personally experienced expulsion from Cordoba for being a Jew. This began a lifetime of exile in which he became established in different nations, only to have to move yet again. In his lifetime he lived in Cordoba, Spain; Fez, Morocco; Acco, Israel; Alexandria and Fustat (Old Cairo), Egypt; and he visited numerous countries in between. As a rabbi, he was fully conversant with the Hebrew scriptures and all the Talmudic writings. A consummate researcher and physician, Maimonides was highly trained and knowledgeable of the writings of Galen, Hippocrates, and Aristotle, the medical writings of ancient Egyptian scholars, and he often quoted the writings of Spanish-Moslem Arabic physicians.^2^,^3^ He carefully examined the medical practice of the people among whom he lived and synthesized a unique practice of medicine that combined what his observations indicated were best for all humanity. Comparing Galen with Maimonides, the famous Arabic poet and physician Alsaid Ibn Sina Almulk wrote: “Galen’s art heals only the body, but Maimonides’ skill heals the body and soul. … When Maimonides arrives, all suffering departs.”^4^

In addition to his major religious works, Maimonides published 10 medical books.^3^ His writings had a strong focus on preventive medicine and physical activity in general but included specific recommendations regarding asthma, hemorrhoids, coitus, intestinal health, nutrition, and more.

### How Maimonides Related to Prior Learning and Knowledge

Maimonides’ comprehensive knowledge converged with his daily experience and practice, leading him to uniquely synthesize a medical perspective that excluded commonly held ideologies that he considered unacceptable. For example, in his medical text *Pirkei Moshe*, Maimonides explains in the introduction that he included only the cures referred to by Hippocrates and Galen and other gentile physicians. “These are the chapters I have gathered, not that I have written them, but I have chosen them, from the works of Galen, … the works of Hippocrates … and I have meticulously investigated them.”5(a) In particular, Maimonides considered Galen to be an “excellent scholar.”5(b)

His meticulous investigation is reflected in *Pirkei Moshe* in a chapter entitled “The Holy War with Galen,” where Maimonides stated that he had several doubts regarding some of Galen’s works and that he did not accept all that Galen said, choosing instead to follow only logical or experimental evidence.5(b) To so strongly reject some of the teachings of Galen, who was referred to at that time as “divine Galen,”^6^ took great courage and confidence, and reflects Maimonides’ personal sense of responsibility to present only what he considered to be proven medical guidelines.

In later years, the son and successor of Maimonides, Rabbi Abraham, wrote that one should not accept the medical teachings of the Jewish sages without investigation and verification. “Anyone who wants to act on these opinions without investigation, and without understanding whether or not the issue is true, is following a path that is forbidden by the Torah and common sense.”^7^

### Importance of Maimonides’ Recommendations for Medical Practice

Maimonides died at the age of 69 in 1204 in Fustat and was buried in Tiberias, Israel. However, as stated above, his impact was far-ranging. It is interesting to note, for example, that a web search for “Maimonides” <AND> “Diet” <AND> “Plan” gives literally hundreds of thousands of hits. There are other recommendations that Maimonides made, based only on observation, that today are researched best practices in medicine, including exercise and concern for clean air.

The importance that Maimonides placed on medicine led him to include a number of chapters, mainly Chapter 4, in his halakhic essay *Mishneh Torah* (Repetition of the Torah) in the “Sefer Madda” (The Book of Wisdom) “Hilchot De’ot” (Laws of Human Dispositions), dealing with preventive medicine and proper health behavior which are the essence of his medical training for healthy people.^8^

His recommendations demonstrated a fundamental understanding of the human body based on the cumulative knowledge of medical science dating back to Hippocrates, Aristotle, and Galen (~460/384 BC, and 129 AD). This must be taken into consideration when considering the recommendations of Maimonides. For example, there was no refrigeration or pasteurization, and the cause of most diseases was unknown. Despite this, what is most interesting is that many of his recommendations remain true today. This is remarkable given the available knowledge and customs of his time.

The aim of this study was to compare Maimonides’ recommendations for healthy nutrition and eating habits in healthy people to recent guidelines.

## METHODS

Recent United States (US),^9^,^10^ Dutch,^11^ and World Health Organization (WHO)^12^ guidelines, and the Mediterranean Diet—which is extremely popular worldwide^13^,^14^—were compared to each other and to those of Maimonides.^8^

## RESULTS

The results for the above-mentioned comparison are summarized in Table 1. Of the 20 dietary recommendations by Maimonides, the USA, Netherlands, WHO, and Mediterranean Diet fully agreed with five, fully disagreed with three, and there was notable limited or full agreement for 12 other recommendations.

**Table 1:** Agreement between Maimonides’ Recommended Dietary Regimen and Contemporary Recommendations.

Dietary Regimen Item	Maimonides^9^*	USA^10^,^11^*	The Netherlands^12^*	WHO^15^*	Mediterranean Diet^13^,^14^*

Bread	Coarse flour with husk Limited: matzot and barley bread	Agree	Agree	Agree	Agree

Fresh fruit	Avoid *all* fruit, even if dried and especially fresh, except for figs, grapes, or dates (fresh or dried) in small amounts	Disagree	Disagree	Disagree	Disagree

Vegetables	Certain types of cucumbers, certain types of zucchini should be eaten first, before the meal, and not together with the main meal Limited: cabbage, leeks, onions, garlic, mustard and radishes Forbidden: truffles, mushrooms	Disagree	Disagree	Disagree	Disagree

Legumes	Limited: fava beans, lentils, chickpeas	Disagree	Disagree	Disagree	Disagree

Meats					

General (lamb/sheep, poultry, beef)	Generally good Avoid fat	Agree	Limited agreement: limit amount of red meat	Agree	Limited agreement: Limit to less than two servings weekly

Processed meat	Extremely harmful: aged/salted/processed meat	Limited agreement: consumption should be limited because of high salt content	Agree	Agree	Limited agreement: less than 1 portion weekly

Fish	Generally restrict	Disagree	Disagree	Disagree^16^	Generally agree: two or more servings weekly (small amounts)

Salted/aged	Avoid	Agree	Agree	Agree	Agree

Nuts	Generally good (especially almonds)	Agree	Agree	Agree	Agree

Eggs	Good	Agree	Limited agreement: limit consumption	Limited agreement^17^	Agree

Milk products					
Milk	Fresh only (less than 24 hours after milking)	Limited agreement: pasteurized only^18^	Limited agreement: allow both raw milk and pasteurized^19^	Limited agreement: low-fat recommended	Limited agreement: low-fat recommendation, yogurts and cheeses preferred

Fresh, sweet, or light cheese	Good	Limited agreement: fat-free or low fat; avoid soft cheeses made from unpasteurized milk in pregnant women	Limited agreement (cheese in general permitted)	N/C	Agree

Hard cheese	Limited	N/C	Limited agreement (cheese in general permitted)	Agree	N/C

Fats	Fats in general considered bad	Partially agree: limit saturated and trans fats	Partially agree: eat less animal fat	Partially agree: reduce fat intake to less than 10% of total energy intake	Partially agree: moderation and special occasions

Olive oil	Good–cleanses the liver and loosens stools	Agree	Agree	Agree	Agree

Butter	Limited use (fresh and clarified)	Agree	Disagree: should be avoided	Agree	Agree

Honey					
For adults	Good	Limited agreement	N/C	Limited agreement	N/C

For children	Bad	Agree^20^	N/C	Agree^21^	N/C

Salt	Recommendations imply strong limitations on salted food	Agree	Agree	Agree	Agree

Alcohol consumption	Permitted Harmful to the young and wholesome for the old	Limited agreement	Limited agreement: avoid, or no more than 1 glass daily	Limited agreement: not recommended for children and pregnant women; limited use by other adults^22^,^23^	Limited agreement: small amount of wine at mealtime

* Unless otherwise indicated within the table, then those references take precedence.

While reviewing Maimonides’ dietary recommendations, several additional recommendations for eating habits were also noted. These were tied so closely to his dietary recommendations that they were also summarized and compared to the contemporary recommendations.

## DISCUSSION

The purpose of this study was to find out how many of Maimonides’ 20 main food recommendations, written more than 800 years ago, are accepted today according to four modern major dietary guidelines. This study showed that only three of Maimonides’ recommendations should be fully rejected. The other 17 recommendations received either full or partial agreement.

The principles of health proposed by Maimonides present proper nutrition as essential for the health of both the body and the mind. In addition, he believed that physical activity was an important factor for maintaining health and emphasized that a person’s duty was to maintain one’s health before illness ever occurred (known in modern times as “preventive medicine”).

Among Maimonides’ medical recommendations were many commonly accepted today worldwide: development of healthy habits to maintain health; preventive medicine; holistic mental health medicine; the need for exercise; orderly and moderate eating; adequate sleep; and personal hygiene.

### Rationale for Specific Food Recommendations by Maimonides

The fact that most of Maimonides’ nutritional recommendations are basically accepted by some of the major authorities worldwide is remarkable. Maimonides based his recommendations on astute observation of his patients throughout decades of medical practice. The only total disagreement between the modern recommendations and Maimonides relates to the consumption of fresh fruits, vegetables, and legumes. Hence it is important to understand his rationale for these recommendations.

When reading his recommendations in De’ot carefully, three particular themes recur throughout: the concern for proper digestion, healthy defecation, and urination. Hence, he suggested the eating of laxative fruits and vegetables first (grapes, figs, mulberries, pears, melons, and certain types of zucchini), while constipating fruits and vegetables were recommended for after the meal, and not in large amounts (e.g. pomegranates, quinces, apples, etc.).8(a) However, at the heart of everything he writes regarding diet is his concern for consistent, lifelong stool consistency that is loose and tends slightly towards diarrhea.

This is a cardinal principle in medicine: Whenever one suffers from constipation or has difficulty moving his bowels, serious diseases will beset him.8(b)

This begs the question, why? Magrill and Sekaran point out that his recommendations help to prevent hemorrhoids.^33^ Trowel discusses at length the importance of soft stool for health.^34^ It is well known today that constipation is often associated with stress, depression, and little physical activity or exercise.^35^ Maimonides makes reference to issues such as mood, exercise, and stress in connection with his dietary recommendations, and it is highly likely that he would have observed lose stool as a sign of healing. In addition, it must be remembered that diet was the only tool in a physician’s box for constipation during the time of Maimonides. Today, with the plethora of medications on the market, dietary considerations have become but one aspect of treating constipation and hemorrhoids. This, in addition to the fact that today there are no problems with food storage, could help explain why modern dietary recommendations for fruits and vegetables disagree with those of Maimonides.

Another possible reason why Maimonides had such a low view of fruits could be that Galen strongly opposed their use. Professor Gamliel suggests that it is possible that people did not wash fruits before eating, and they did not have the soaps and disinfectants used today. Hence, the fruits could have had a greater number of bacteria, microbes, parasites, etc., which led to a stronger intestinal reaction in Galen and his patients.^36^

With regard to legumes, Maimonides does not write specific reasons for referring to “horse-beans” (known today as fava beans), lentils, chickpeas, and other legumes as harmful and something to be avoided or eaten sparingly. However, today fava beans are recognized to be harmful and even life-threatening for certain people suffering from G6PD deficiency. This condition is transmitted genetically and is more likely to be found in people of Middle Eastern, Kurdish, or Sephardic Jewish descent.^35^

### Other Health Recommendations of Maimonides

Maimonides also made additional health recommendations. Table 2 looks at some of those, which, in some way, are connected to eating habits in his writings. Here, the agreement between certain items is remarkable, albeit not in the dietary guidelines of most of the compared recommendations (i.e. exercise, amount of food consumed, food odor, and exercise after eating). Also noteworthy is the silence on many issues, i.e. seasonal eating, water intake at mealtime, stool quality, timing of eating, and sequence of foods eaten—all of which could well bear further investigation as they relate to healthy eating habits.

**Table 2:** Agreement between Maimonides’ Recommended Eating Habits and Contemporary Recommendations.

Eating Habit	Maimonides^9^*	USA^10^,^11^*	Netherlands^12^*	WHO^15^*	Mediterranean Diet^13^,^14^*
Exercise	Regular exercise AND not eating to satiation AND “loose bowels” are recipe for health–even if person eats harmful foods	Agree^24^	Agree but not in dietary guidelines^25^	Agree but not in dietary guidelines^26^	N/C
Air quality	Avoid polluted air^27^	Agree^28^	Agree^29^	Agree^30^	N/C
Water at mealtime	Small amount mixed with wine during the meal†	N/C	N/C	N/C	N/C
Stool quality	Soft is preferable	N/C	N/C	N/C	N/C
Food odor	Avoid foods with putrid odor	N/C	N/C	Agree^31^	N/C
Timing of eating food	Only eat after true hunger	N/C	N/C	N/C	N/C
Sequence of foods	Lightest foods first, foods difficult to consume last	N/C	N/C	N/C	N/C
Amount of food	Not to fullness (3/4 satisfied)	Agree: limit calories for daily intake; do not eat if not hungry^32^	Agree: limit calories for daily intake	Agree: limit calories for daily intake	Agree: limit calories for daily intake
Exercise after eating	No heavy exertion, walking, strolling until food is digested	Partial agreement: no exertion after eating^33^	N/C	N/C	N/C
Seasonal eating	Specific foods should be eaten only in summer or winter, but not both	N/C	N/C	N/C	N/C

* Unless otherwise indicated within the table, then those references take precedence.

† Maimonides made several other recommendations regarding how water should be consumed, including 30 minutes before or after a meal; 1 hour after meal, and drinking a glass of water with each meal and between each meal.

N/C, no commentary in the guidelines.

The issue of clean air was included in this table since Maimonides was one of the first in his time to understand the implications of air quality on health. Noteworthy is that this issue has only become an important health concern in the last century.

Many of Maimonides’ recommendations were revolutionary in his time, although they are common knowledge today. For example, exercising, not consuming foods with a foul odor, and not eating too much in general fall into the realm of common knowledge. People involved in sports are well aware of the recommendation to avoid heavy exercise 1–2 hours after eating. However, this was new information for the patients who came to Maimonides, and testifies to his skilled observational and inductive abilities. Only a few of the items listed in Table 2 are included in present-day established dietary and health recommendations.

## CONCLUSION

More than 800 years later, Maimonides’ dietary recommendations remain relevant and pertinent to the twenty-first century. Modern technological advances continue to confirm the basic principles of his original work. In addition, his advice regarding the quality and quantity of food, exercise, air quality, and sobriety not only continue to be applicable today, but are considered to be common sense by many.

For today’s practicing physicians, these findings should be a strong reminder that knowledge and modern technologies are not enough for patient care; physicians must continue to focus on honing their observational, deductive, and inductive skills for the benefit of patients. Courage is also required, to oppose applicable modern practice when a better way can be found.

Indeed, the personal promise that Maimonides gave to anyone who followed his instructions has relevance to this day:

Whoever conducts himself in the ways which we have drawn up, I will guarantee that he will not become ill throughout his life, until he reaches advanced age and dies. He will not need a doctor. His body will remain intact and healthy throughout his life.8(c)

## Abbreviations

US: United States
WHO: World Health Organization
